# Bioengineered Nisin Derivative M17Q Has Enhanced Activity against *Staphylococcus epidermidis*

**DOI:** 10.3390/antibiotics9060305

**Published:** 2020-06-06

**Authors:** Ellen Twomey, Colin Hill, Des Field, Maire Begley

**Affiliations:** 1 Department of Biological Sciences, Cork Institute of Technology, T12 P928 Cork, Ireland; e.twomey@mycit.ie; 2 School of Microbiology, University College Cork, T12 YN60 Cork, Ireland; c.hill@ucc.ie; 3 APC Microbiome Ireland, University College Cork, T12 YN60 Cork, Ireland

**Keywords:** bacteriocin, antibacterial peptide, bioengineered peptide, nisin, *Staphylococcus epidermidis*, medical device related infections, biofilm

## Abstract

*Staphylococcus epidermidis* is frequently implicated in medical device-related infections. As a result of this, novel approaches for control of this opportunistic pathogen are required. We examined the ability of the natural peptide nisin A, produced by *Lactococcus lactis*, to inhibit *S. epidermidis.* In addition, a bank of 29 rationally selected bioengineered *L. lactis* strains were examined with the aim of identifying a nisin derivative with enhanced antimicrobial activity. Agar-based deferred antagonism assays revealed that wild type nisin A inhibited all 18 *S. epidermidis* strains tested. Larger zones of inhibition than those obtained from the nisin A producing *L. lactis* strain were observed for each derivative producer against at least one *S. epidermidis* strain tested. Six derivative producing strains, (VGA, VGT, SGK, M21A, M17Q, AAA), gave larger zones against all 18 strains compared to the wildtype producing strain. The enhanced bioactivity of M17Q was confirmed using well diffusion, minimum inhibitory concentration (MIC) and a broth-based survival assays. Biofilm assays were performed with plastic microtiter plates and medical device substrates (stainless-steel coupons and three catheter materials). The presence of nisin A significantly reduce the amount of biofilm formed on all surfaces. M17Q was significantly better at reducing biofilm production than nisin A on plastic and stainless-steel. Finally, M17Q was significantly better than nisin A at reducing bacterial numbers in a simulated wound fluid. The findings of this study suggest that nisin and bioengineered derivatives warrant further investigation as potential strategies for the control of *S. epidermidis.*

## 1. Introduction

*Staphylococcus epidermidis* is a habitual member of the human commensal microbiota [1,2]. While most frequently found on the skin, this Gram-positive, coagulase negative organism has also been isolated from the mucous membranes of the nares [2]. Although benign when present on the skin of healthy individuals, *S. epidermidis* has been classed as an opportunistic pathogen [1,3]. Lee et al. have described it as a “formidable nosocomial pathogen,” due to its rapid spread, the development of resistance, its threat to immunocompromised individuals [1,3]. The ability of *S. epidermidis* to form biofilms on both biotic and abiotic surfaces and invade the body via postoperative skin wounds, complicates the recovery process and increases patient suffering. Easily transferred due to its ubiquitous presence on the skin, the production of an exopolysaccharide matrix permits planktonic *S. epidermidis* cells to adhere to the surfaces of indwelling medical devices, such as intramedullary pins, prosthetic devices, for example, artificial hips, catheters and cerebrospinal shunts [4,5]. Once the organism has invaded or been implanted into the body of the patient, development of a slime layer establishes a persistent source of infection within the body, where the most viable treatment option is the complete removal and replacement of the device [4,6]. By extracting and replacing contaminated devices such as prosthetic joints, pacemakers or shunts, afflicted individuals potentially face a secondary invasive surgery, as well as unnecessary pain and suffering. Such removals are coupled with a course of antibiotics to ensure the infection is cleared and to promote recovery [3]. However, a 2018 summary report released by the European Centre for Disease Prevention and Control remarks on the rapid emergence and spread of Multidrug Resistant *S. epidermidis,* (MRSE) [3]. This states that the efficacy of previously used successful drugs such as trimethoprim, fusidic acid, clindamycin and fluoroquinolones are in decline, with cases of resistance against the first line of defense, vancomycin, also being observed [3]. With the looming threat of resistant infections and a depleted antibiotic discovery pipeline, alternate treatment routes are being investigated.

Bacteriocins are ribosomally synthesized peptides that are produced by some bacteria; however, they usually operate selectively against closely related strains [7,8]. Nisin is a 34-amino acid bacteriocin produced by *Lactococcus lactis* with antimicrobial activity against a number of Gram-positive pathogens such as Methicillin Resistant *Staphylococcus aureus* (MRSA), streptococci and Vancomycin Resistant Enterococci [8]. Nisin exerts its antimicrobial effects by binding to a highly conserved component of the bacterial cell wall, lipid II [8,9]. By interfering with this integral element, it causes the formation of pores and prevents cell wall synthesis, triggering lysis and cellular death [8,9]. Nisin has been approved by both the United States Food and Drug Administration (FDA) and the European Food Safety Authority (EFSA) for use as a food preservative in over 70 countries and is “generally regarded as safe” (GRAS) [10]. In recent years, studies have revealed nisin to be effective against an array of clinically relevant Gram-positive pathogens, including *Listeria monocytogenes* and drug resistant *Staphylococcus aureus* [8,11]. While not currently used in clinical settings, bacteriocins are demonstrating their capabilities at inhibiting the spread of infections in murine and other animal models. For example, nisin has been applied to wound dressings and catheters used on or in, cows undergoing routine surgical procedures and successfully reduced the likelihood of infections [12]. The bacteriocin lacticin 3147 has been found to inhibit the spread of *Staphylococcus* infections in a murine model [13].

Several naturally occurring variants of nisin have been found to be produced by *L. lactis*, with one *Streptococcus* species, (*Streptococcus uberis*) also possessing genes for another version of the peptide [14,15]. All variants discovered have identical modifications as the initially characterized nisin peptide nisin A but differ in their amino acid compositions. For example, nisin Z has histidine at position 27 in place of asparagine. Nisin Q contains a more varied sequence which differs by four amino acids, (valine at position 15, an asparagine at position 17, leucine at position 21 and another valine at position 30) [16,17]. All naturally occurring peptides are capable of inhibiting the growth of clinically relevant Gram-positive bacteria in agar based deferred antagonism and agar well diffusion assays [15,16,17]. In addition to the occurrence of natural variants of nisin, the gene encoded nature of the peptide has permitted extensive bioengineering with the hopes of creating novel nisin peptide derivatives with more desirable properties such as increased solubility, stability and antimicrobial activity. In our laboratory, we have employed site directed and site saturated mutagenesis approaches to generate an extensive and diverse bank of *L. lactis* strains that produce bioengineered nisin derivatives. These include strains genetically engineered to produced mutated versions of the wildtype nisin A peptide [8,14,18,19]. Several of the generated strains have displayed enhanced antimicrobial activity against selected pathogens when compared to the wildtype. For example, the bioengineered peptide I4V demonstrated a two to four-fold increase in activity (as determined by minimum inhibitory concentration assays) against three strains of *Staphylococcus pseudintermedius* a Gram-positive pathogen responsible for skin and soft issue infections in both humans and animal species [20]. Similarly, the bioengineered peptides M21A and M21V demonstrated a two-fold increased activity compared to the wildtype nisin A against the foodborne pathogen *L. monocytogenes* [21]. 

The ability of nisin to inhibit *S. epidermidis* has been reported. Davison et al. investigated nisin along with several other biocides against one *S. epidermidis* strain (RP62A) that is a strong biofilm producer [22]. The test was performed using capillary flow cells and confirmed that the bacteriocin possessed antimicrobial activity against the target, *S. epidermidis* RP62A [22]. Ghiselli et al. have studied the effect of nisin against *S. epidermidis* (two strains) graft infections in mice. Pre-treatment of the prosthetic graft material Dacron with nisin reduced bacterial counts by four logs [23]. Here we investigated the ability of nisin A to inhibit a larger number of *S. epidermidis* strains. The second objective was to subsequently interrogate a rationally selected bank of 29 *L. lactis* strains that produce bioengineered nisin derivatives (selected based on their ability to inhibit other Gram positive pathogens in separate studies in our laboratory) with the aim of identifying a derivative peptide with enhanced ability to inhibit *S. epidermidis*.

## 2. Results

### 2.1. Agar Based Deferred Antagonism and Well Diffusion Assays

**Figure 1:**
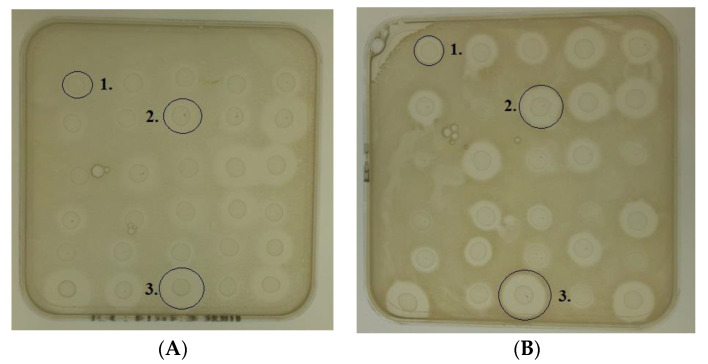
Representative pictures of the agar based deferred antagonism assays that were performed with a wildtype producing *L. lactis* strain and 29 *L. lactis* strains that produce bioengineered nisin A derivatives. These strains were spotted onto GM17 agar and grown at 30 °C overnight after which they were overlaid with tryptone soy agar (TSA) inoculated with (**A**) *Staphylococcus epidermidis* 28 and (**B**) *Staphylococcus epidermidis* 53. Overlaid plates were incubated for 18 h at 37 °C. Zones of inhibition from the (1) nisin A producing strain (2) M21V producing strain and (3) M17Q producing strain are circled.

The ability of a wildtype nisin A-producing *L. lactis* strain and a bank of 29 *L. lactis* strains each producing a different variant of nisin A were examined for their ability to inhibit a collection of 18 *S. epidermidis* strains by performing agar based deferred antagonism assays (Figure 1). Zones of inhibition were observed for the wildtype nisin A producer against all of the 18 *S. epidermidis* strains with representative zone sizes ranging from 9.8 to 16.3 mm (Table 1). With the exception of the I4V producing strain against *S. epidermidis* 1, zones of inhibition were also observed for all of the 29 nisin derivative producers against all 18 *S. epidermidis* strains. Each nisin derivative producing strain produced at least one enhanced zone against a member of the bank of 18 strains when compared to the wildtype. Not all *S. epidermidis* strains were impacted equally, despite all being of the same species. For example, M21V had enhanced activity against 16 of the 18 strains used but against strains 26 and 1 it was found to possess activity equal to or less than nisin A. In all, it was noted that two derivative producing strains gave larger zones of inhibition than the wildtype against 17 *S. epidermidis* strains and six bioengineered derivative producing strains gave larger zones than the wildtype against all 18 *S. epidermidis* strains tested. Of these six (VGA, VGT, SGK, M21A, M17Q and AAA), the M17Q and AAA producers gave the largest zones of inhibition. Representative zone sizes ranging from 14 to 21 mm were observed for the AAA producing strain and representative zone sizes ranging from 13.1 to 21 mm were observed for the M17Q producing strain. The AAA derivative peptide was previously described as part of a study to investigate nisin derivatives containing small chiral amino acids within the hinge region [24]. This hinge region connects the N terminal of the nisin peptide, which contains one lanthionine and two (β-methyl) lanthionine rings (A, B and C), to the C terminal which is composed of intertwined rings, D and E, (see Figure S1). Notably, although it exhibited enhanced bioactivity through deferred antagonism assays, the AAA derivative exhibited enhanced specific activity against just one of four strains it was tested against [24] revealing that in many cases it would appear that enhanced bioactivity may be attributable to enhanced diffusion through complex media, a phenomenon previously reported by Rouse et al., 2012 [25]. Given that M17Q represents a new enhanced derivative at a location that has not previously been identified by NMR as a ‘hotspot’ critical for the cellular adaptability of nisin [26], it was decided to focus on M17Q for the remainder of the study. In the M17Q peptide the methionine (M) at position 17, (i.e., M17), in the wildtype nisin is replaced with a glutamine (see Figure S1). For the M21V producing strain, larger zones of inhibition were obtained for 16 of the 18 *S. epidermidis* strains when compared to the wildtype producing strain. M21V has been the subject of a number of studies to evaluate its efficacy against a wide range of pathogenic bacteria, including *L. monocytogenes* MRSA, hVISA and *C. difficile* so it was decided to include it in subsequent experiments for comparison purposes [11,18,19,27].

### 2.2. Agar Well Diffusion Assays, Minimum Inhibitory Concentration Assays and Kill Curve

**Figure 2:**
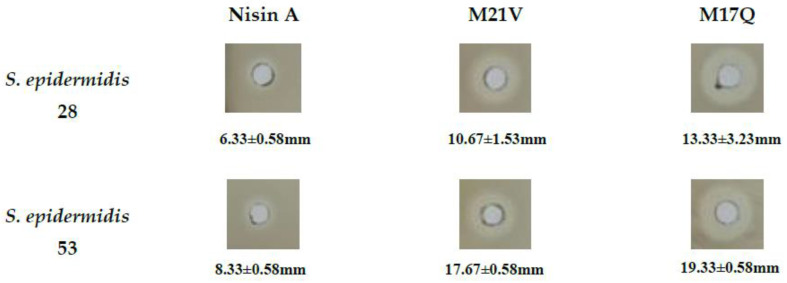
Images are representative of the average zones of clearing obtained when 30 μL of a 30 μM stock of each peptide was added to well in Tryptone soya agar. Plates were incubated at 37 °C overnight.

**Figure 3:**
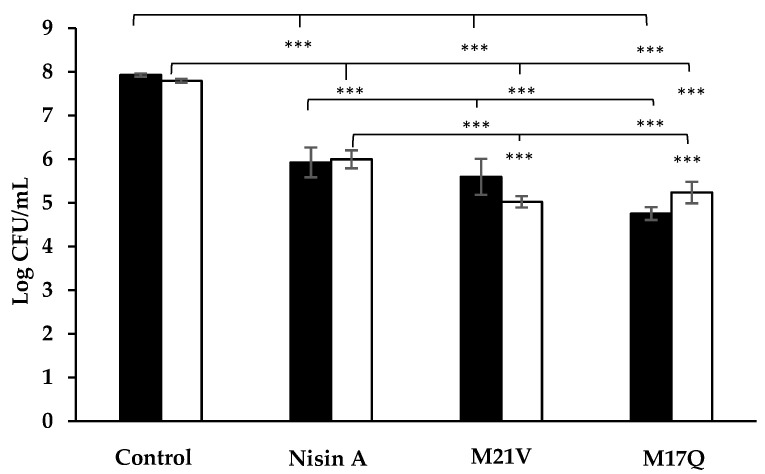
Counts of *S. epidermidis* 28 (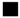) and *S. epidermidis* 53 (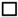) after 1 h incubation in tryptone soya broth supplemented with 3.75μM nisin A, M21V or M17Q peptide. (*** = *p* < 0.001).

Nisin A, M21V and M17Q peptides were purified from the relevant *L. lactis* producing strains by HPLC, (see Figures S2 and S3). The ability of the three peptides to inhibit two representative *S. epidermidis* strains (28 and 53; chosen as they were strong biofilm formers; see later) was examined using three different assays—agar well diffusion assay, MIC assay and a broth-based kill curve. As can be seen in Figure 2, while all three peptides produced distinct zones of inhibition in the well diffusion assays, the zones produced by the derivative producing strains appeared to be enhanced when compare to the wildtype. Using a standard broth-based MIC test, it was observed that in the case of both *S. epidermidis* 28 and 53, M17Q and M21V had a two-fold reduction in MIC values when compared to the wildtype nisin A peptide (see Table 2). The broth-based assay revealed that the concentration of nisin A employed resulted in ~2 log reduction in the numbers of both *S. epidermidis* strains after the 1 h incubation time. The same concentration of M21V caused ~2.5 log reduction in *S. epidermidis* 28 and ~3 log reduction in *S. epidermidis* 53 numbers. A ~3 log reduction of both *S. epidermidis* strains was observed for M17Q, the results of which are presented in Figure 3. 

### 2.3. Inhibition of Biofilm Formation on Plastic

**Figure 4:**
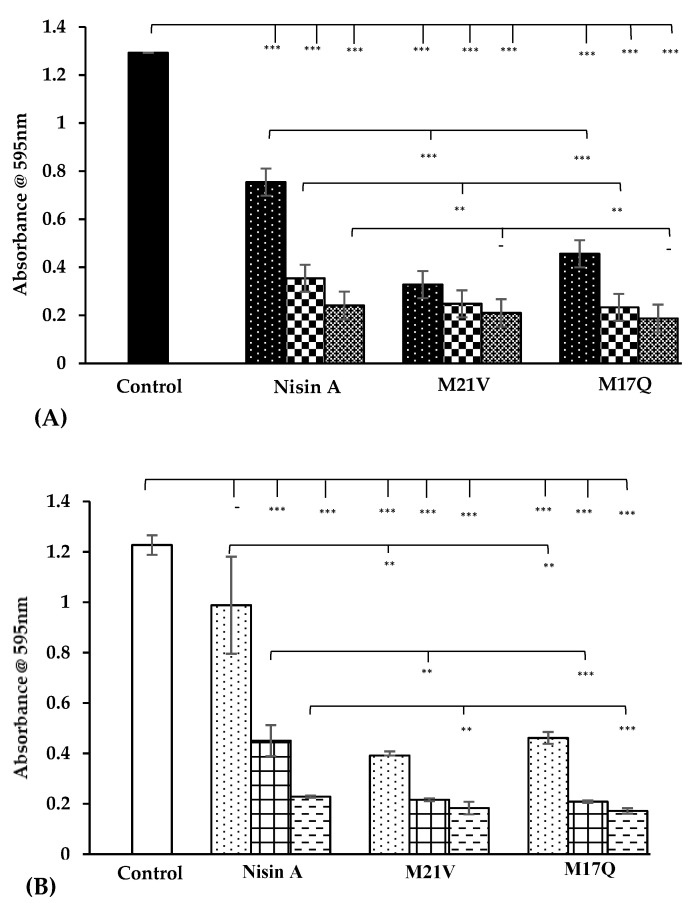
Absorbances of biofilm formed by (**A**) *S. epidermidis* 28 and (**B**) *S. epidermidis* 53 on plastic microtiter plates in the presence 1.875 μM, (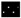, 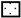), 3.75 μM, (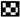, 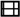) and 7.5 μM, (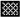, 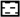) nisin A and derivative peptides, M21V and M17Q. Control shows the amount of biofilm produced in the absence of nisin, (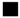, 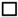). *** denotes *p* < 0.001, ** denotes *p* < 0.005, * denotes *p <* 0.05 and “-”denotes no statistical significance.

The ability of the 18 *S. epidermidis* strains to adhere to and form biofilm on plastic microtiter plates was analyzed using an established method [28]. All 18 strains were found to be capable of forming biofilms to varying degrees on this surface (data not shown). Strains *S. epidermidis* 28 and *S. epidermidis* 53 can be considered strong biofilm formers also based on this protocol. A comparison of the absorbances obtained from the negative controls and the nisin A wildtype peptide, revealed a significant reduction in biofilm formation at all three concentrations of nisin that were tested (1.875, 3.75 and 7.5 μM) (Figure 4). When the antibiofilm effects of M21V and M17Q were compared to the wildtype a significantly enhanced reduction in absorbance was observed against both *S. epidermidis* 28 and 53 at both 1.875 and 3.75 μM. Against strain 53 at 7.5 μMol, the antibiofilm forming capabilities of the derivatives were significantly enhanced compared to nisin A, (*p* value ≤ 0.05). However, against *S. epidermidis* 28 at 7.5 μM, the absorbances of the wildtype and the derivative peptides were similar. Overall, M17Q was seen to negatively affect biofilm formation similar to M21V that is, enhanced compared to nisin A.

### 2.4. Biofilm Inhibition on Stainless Steel by M17Q

**Figure 5:**
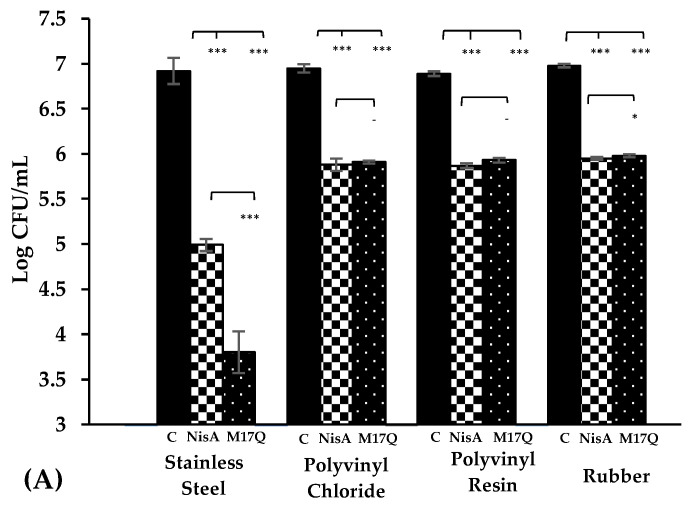

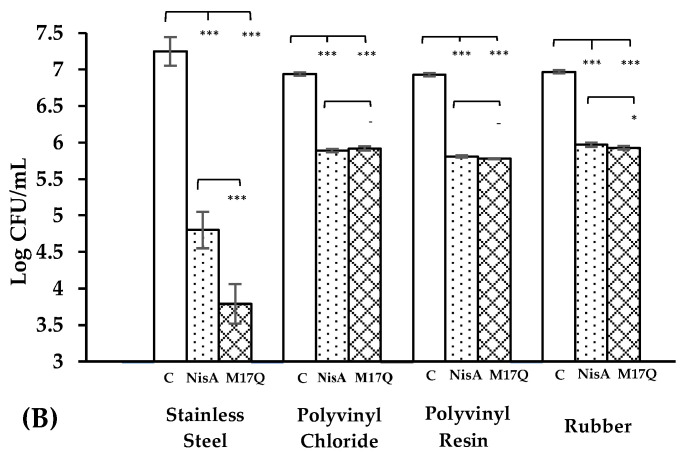
Colony forming units recovered from (**A**) *S. epidermidis* 28 and (**B**) *S. epidermidis* 53 on stainless-steel and catheter materials in the absence of nisin (C, 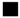, 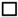), the presence of nisin A at 3.75 μM, (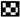, 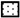) and derivative peptide M17Q at 3.75 μM, (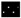, 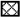). *** denotes *p* < 0.001, ** denotes *p* < 0.005, * denotes *p <* 0.05 and—shows no statistical significance.

The remaining experiments focused on M17Q. Assessing the formation of biofilm on stainless steel, (a material that can be used in artificial hips), was performed by calculating CFU/mL recovered from scrapings taken off the surface of stainless steel coupons following 24 h incubation with *S. epidermidis*. The negative controls used in this experiment, *S. epidermidis* strains in broth without nisin, confirmed that *S. epidermidis* 28 and 53 could form substantial biofilms and adhere to the surface of the stainless steel coupons (Figure 5). By comparing these controls to the strains incubated with nisin A, it was confirmed that the wildtype peptide could interfere with biofilm formation on this surface type. Nisin A caused ~2-log reduction in strain 28 and a 1.5 log reduction in strain 53 counts, in both instances the reduction was found to be statistically significant (*p* < 0.001). M17Q also impacted *S. epidermidis* biofilm formation in a significant way, causing an approximate 3-log lower recovery of both *S. epidermidis* strains when compared to the control. This log difference in numbers compared to nisin A was deemed statistically significant (*p* < 0.01).

### 2.5. Biofilm Assay on Various Catheter Materials

As with the stainless steel coupons, the degree of biofilm formation on catheters was assessed by determining bacterial counts recovered from the surface of each material type. The catheter tubes made of rubber, polyvinyl chloride and polyvinyl resin, were cut into even 2 cm pieces that were hollow on the inside. The negative controls, (catheter pieces incubated with *S. epidermidis* culture in the absence of nisin), confirmed that strains 28 and 53 could form biofilm and adhere to the three different surface types (Figure 5). When evaluating the impact of nisin A on *S. epidermidis* biofilm formation on catheters, it was observed that the wildtype peptide caused a single log reduction for both strain 28 and 53 on all material types when compared to the control. These decreases in CFU/mL were calculated to be statistically significant (*p* < 0.001). M17Q also caused a single log reduction in both strains, on all material types tested. This log reduction was also found to be statistically significant compared to the control (*p* < 0.001).

### 2.6. Testing the Efficacy of Nisin A and M17Q in a Simulated Wound Fluid

**Figure 6:**
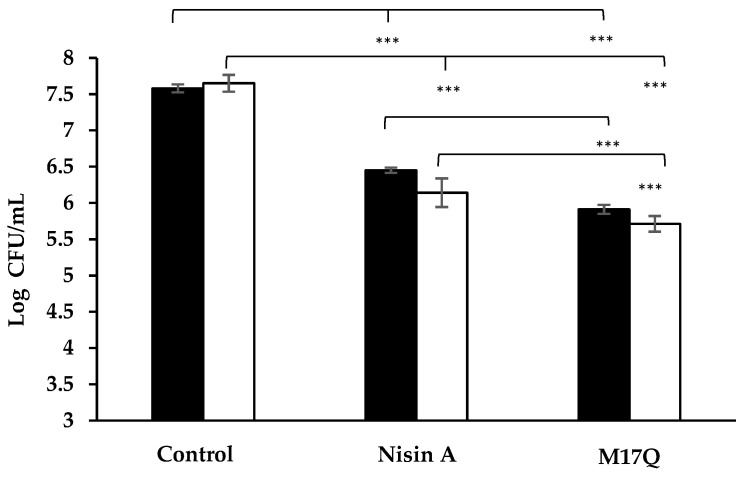
Kill curve performed in a simulated wound fluid where *S. epidermidis* 28 (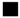) and *S. epidermidis* 53 (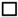) were incubated for 1hr at 37 °C with nisin A and M17Q at a concentration of 3.75 μM. *** denotes *p* < 0.001, ** denotes *p* < 0.005, * denotes *p <* 0.05 and “-” denotes no statistical significance.

*S. epidermidis* 28 and 53 were incubated in a simulated wound fluid with or without nisin to determine if antimicrobial activity was retained in this setting. The negative controls, *S. epidermidis* strains cultured in the absence of nisin, verified that the viability of strains was not affected by exposure to the wound fluid (Figure 6). The addition of nisin A at a concentration of 3.75 μM resulted in ~1 log reduction in counts of *S. epidermidis* 28 and a reduction of ~1.5 logs for *S. epidermidis* 53 in a simulated wound fluid. The same concentration of M17Q caused ~2 log reduction in counts of both strains in a simulated wound fluid. While both nisin A and M17Q caused a statistically significant reduction in bacterial numbers (*p* < 0.001), the results obtained for M17Q were significantly lower than those obtained for nisin A (*p <* 0.001 for strain 28, *p <* 0.05 for strain 53). 

## 3. Discussion

*S. epidermidis* is an opportunistic pathogen with an ability to form biofilms on medical devices including cerebrospinal shunts, catheters, artificial hips and pacemakers [4,5]. Its ubiquitous presence on skin means transfer and contamination of indwelling devices is common [29]. The appearance of MRSE further enhances the threat posed, as conventional antibiotic treatments are losing efficacy [3]. This increase in antibiotic resistance coupled with the expected rise in medical device interventions due to an increasing elderly population mean that alternative strategies to control *S. epidermidis* are required. A major focus of research carried out in our laboratory is on the identification and characterization of a group of natural microbially produced antimicrobial peptides called bacteriocins. While traditionally researched for their applications as food preservatives it has become increasingly evident in recent years that bacteriocins show promise as therapeutic agents against medically important pathogens including antibiotic resistant bacteria [30]. Of particular interest is the fact that bacteriocins are gene encoded as this permits the generation of bacteriocin variants which may possess more desirable properties. We have extensively bioengineered the bacteriocin nisin A and have assembled a large bank of nisin A derivative producing *L. lactis* strains. A sub-bank of 29 rationally selected derivative-producing strains were the focus of the current study.

Agar based deferred antagonism assays revealed that the wildtype nisin A producing strain inhibited all of the 18 *S. epidermidis* strains that were tested. Six of the 29 nisin derivative producers (VGA, VGT, SGK, M21A, M17Q, AAA) produced significantly larger zones of inhibition that the wildtype against all 18 *S. epidermidis* strains. The remaining nisin derivative producers gave zones of various sizes; with enhanced activity against a large portion but not the entire set of *S. epidermidis* strains used. The largest zones were observed for AAA and M17Q producers. We have previously reported that the AAA derivative exhibits enhanced diffusion properties [24]. Indeed, we have also described a similar finding for the hinge derivatives NAK and SVA [25]. Consequently, AAA was not considered further and subsequent experiments focused on confirming the enhanced bioactivity of M17Q. Three separate assays were employed and a well characterized nisin derivative M12V that has enhanced specific activity was included in these experiments for comparative purposes [11,18]. A well diffusion assay revealed that the zones obtained with purified M17Q peptide were significantly increased compared to those obtained for nisin A. MIC assays demonstrated that the MIC value of nisin A obtained for *S. epidermidis* was comparable to the MIC obtained for *L. monocytogenes*, the pathogen that is a primary target of nisin in the food industry [18]. MIC assays also revealed that there was a two-fold reduction in the MIC of M17Q. A broth-based survival assay showed that there was a significant reduction in bacterial counts following exposure of bacteria to M17Q in broth when compared to nisin A (i.e., there was up to 1.5 logs greater reduction in bacterial numbers in the presence of M17Q compared to wildtype nisin A). Altogether, the results confirm the enhanced activity of M17Q against *S. epidermidis*. Interestingly, it was a derivative at methionine 17 that provided the first evidence that nisin Z could be enhanced against particular targets when nisin Z M17Q/G18T demonstrated increased activity against a limited number of non-pathogenic strains (*M. flavus, S. thermophilus*) [14]. The basis of enhanced activity is currently unknown and we do not wish to speculate. However the amino acid substitution of glutamine for methionine at position 17 is located within C ring and it has been shown that this ring can adopt a number of conformations suggesting that this part of the peptide is involved in very specific interactions or may confer unique membrane-interacting properties upon the molecule. As the C ring is vital for the antimicrobial activity of nisin, any failure in its formation would result in an inactive peptide. Mass spectrometry analysis of the peptide confirmed the substitution of methionine, (131 amu), for glutamine, (128 amu) in a decrease of 3amu in the total mass of the peptide. The mass obtained for the derivative suggest that there were no changes to dehydration or cyclization compared to the wildtype peptide but this remains to be confirmed experimentally. 

Biofilms pose a growing threat as due to the nature of the exopolysaccharide and the slow growing state of cells, conventional antibiotics have difficulty crossing the slime layer and eradicating the organisms it protects [31,32]. The slow penetration of the biofilm by antibiotics can expose the cells within to sublethal concentrations and permitting them to develop resistance [32,33]. It has also been noted that once formed, antibiotics are incapable of removing a biofilm [34]. For this reason, prevention of biofilm formation is preferred rather than trying to treat the infection [34]. But with growing levels of resistance reducing the available options for treatment each year, the need for viable alternatives becomes more pertinent. The ability of nisin A to affect biofilm formation by several pathogens such as *L. monocytogenes, S. aureus and S. pseudintermedius* has been investigated and it has been shown that nisin A can impair biofilm formation as well as exhibit bactericidal activity as a result of its ability to access even the deepest part of a biofilm matrix [22,35]. However, it cannot remove previously established biofilms [21,36] unless it is used in combination with other antimicrobials [37]. To our knowledge, the only study in the literature that has examined the effect of nisin A exposure on biofilm formation by *S. epidermidis* is the study of Davison et al. where the authors only investigated its potential to remove established biofilms. While nisin A was shown to penetrate the biofilm, it did not cause any loss in biomass [22]. Taking these previous studies into account it was decided to focus our experiments on biofilm prevention rather than removal. 

Initial assays demonstrated that the *S. epidermidis* strains formed strong biofilms on plastic surfaces. When the experiments were repeated in the presence of nisin A, biofilm formation was significantly impaired. Biofilm production was further impaired in the presence of either M21V or M17Q. In previous biofilm studies in our laboratory with M21V against *S. aureus* the results obtained were similar to those obtained for the wildtype nisin A [36]. Therefore, in addition to reporting the enhanced anti-biofilm formation properties of M17Q, the enhanced anti-biofilm action of M21V is also a novel finding. 

Similar biofilm prevention experiments were then subsequently carried out using medical device-related materials. *S. epidermidis* has frequently been implicated in infections on medical devices made from these materials, for example, artificial hips (stainless steel) and catheters, (PVC, PVR and rubber). When stainless steel was employed as a substrate, nisin A reduced the number of *S. epidermidis* cells that were recovered compared to the control. While nisin A has previously been shown to reduce biofilm formation by *L. monocytogenes* on stainless steel [38], to our knowledge the ability of nisin A to affect biofilm formation by *S. epidermidis* on stainless steel has not previously been reported. The presence of M17Q caused a further impairment in biofilm production compared to the wildtype nisin A. 

Bower et al. assessed the in vitro and in vivo capacity of nisin A to hinder or prevent the growth of *S. aureus*, *S. epidermidis* and *S. faecalis* on Teflon intravenous catheters and PVC tracheotomy tubes [12]. The in vitro examination of Teflon and PVC revealed that when the tubes were incubated with overnight cultures of the selected pathogens for one hour and then placed in nutrient both, the bacteria showed good growth [12]. However, the same strains were noted to grow to a much lesser extent when the tubing was treated with the peptide [12]. The in vivo test, (which entailed inserting the tubing and catheters into animals prior to routine surgical procedures), showed that the skin around the insertion site of nisin treated intravenous Teflon catheters did not become infected, despite being left in for a week [12]. The same was observed for the nisin treated PVC tracheotomy tubes [12]. In our study, three catheter materials were exposed to *S. epidermidis* strains for 24 h in the absence and presence of nisin A or M17Q peptides. Similar to the experiments performed on stainless steel, a significant difference was observed between nisin A and the control. However, it was noted that while nisin A caused up to a 2.5 log reduction in bacterial numbers on stainless steel, it only caused a one log reduction on the catheter materials. Results obtained for M17Q were similar to those obtained for nisin A showing that M17Q did not possess enhanced anti-biofilm ability on the catheter materials under the conditions tested.

Implanted medical devices are not the only cause of *S. epidermidis* related ailments, as this resident of the skin microbiome can also enter the body or establish a site of infection through breaks in the skin like surgical incision sites, burns and other general abrasions [3,39]. It was for this reason that kill curves in a simulated wound fluid were performed to determine whether nisin A and its derivative could maintain their antimicrobial characteristics in a medium representative of the environment of a skin wound. A study conducted in 2016 by Van Staden et al. observed through bioluminescent imaging that the number of *S. aureus* cells in an induced soft tissue wound declined significantly when exposed to the nisin peptide [40]. Although conducted in vitro, our study did find that nisin A and M17Q could inhibit the growth of the selected *S. epidermidis* strains in this alternative media, causing significant decreases in viable cells. Enhanced activity from the derivative peptide was also maintained despite the change from broth to a simulated wound fluid, as it produced an approximate one log greater reduction in bacterial numbers than the wildtype nisin A peptide against *S. epidermidis* 28 and 53. 

To date, nisin resistance has not been observed or reported in *S. epidermidis* strains. Alongside this, nisin A has also been found to promote wound healing, without any observable cytotoxic effects in mammalian cells when tested in animals [12,23,41]. This suggests that nisin A and other nisin derived peptides could be viable treatment options in the fight against and prevention of, medical device associated infections in the future. Nisin A, like many other antimicrobial agents, is not very effective at removing pre-established biofilm due to the nature of the slime layer structure [20]. It is for this reason that prevention rather than removal is preferable. By coating the nisin A or M17Q to the surfaces of medical devices prior to implantation, the peptides could be used as a pre-emptive defense against invading *S. epidermidis* cells. By removing the opportunity for a bacterium to bind to a nisin-coated surface, an infection could be prevented. Peptides such as M17Q could potentially improve patient well-being and reduce treatment costs. By opting to use these peptides in place of conventional antibiotics, the issue of damaging the resident microbiota, developing resistant infections and extended treatment time due to resistance related complications could be also be circumvented. Infections brought about by skin-dwelling *S. epidermidis* entering post-operative wounds or other abrasions could also be potentially be limited through the use of nisin variant-based ointments that could be applied topically. 

## 4. Materials and Methods

### 4.1. Bacterial Strains

The biofilm forming strains of *S. epidermidis* were obtained from the Cork Institute of Technology Culture Collection, having originally being isolated from the blood of patients at Cork University Hospital. All *Staphylococcal* strains were grown in tryptone soy broth (TSB) or tryptone soy agar, (TSA), (Sigma Aldrich, Steinheim, Germany) and grown at 37 °C for 16–18 h. The TSB and TSA were supplemented with 1% glucose (Sigma Aldrich, Steinheim, Germany), when performing biofilm assays. *Lactococcus lactis* NZ9800, a non-nisin producing strain with pDF05 (a pCI372 vector containing the *nisin A* insert) was cultured to harvest the wildtype peptide [19]. This strain will be referred to as the nisin A producing strain. The nisin A and nisin derivative producing strains were all obtained from the University College Cork Culture Collection and selected based on their ability to inhibit a range of Gram positive pathogens in separate studies in our laboratory [11,18,19,24,27,42,43]. *L. lactis* strains were grown as described by Field et al. [19].

### 4.2. Agar Based Deferred Antagonism Assays

In order to determine whether the nisin producing *L. lactis* strains had antimicrobial activity against the bank *S. epidermidis* strains, deferred antagonism assays were performed following the protocol described by Field et al. [42]. The *L. lactis* strains were cultured for 16 h at 30 °C in GM17 broth. 5 μL of each strain was spotted onto GM17 agar and incubated overnight at 30 °C. Following this incubation period, the spots were UV treated in a CL-1000 Ultraviolet Crosslinker for 30 min. 10 mL volumes of sloppy TSA (at 0.75% *w*/*v* agar), was prepared and inoculated individually with 1% of an overnight culture of each *S. epidermidis*. The spotted plates were overlaid with the inoculated agar and incubated overnight at 37 °C. Zones of inhibition in the overlaid agar, indicative of antimicrobial activity, were measured using Vernier calipers, (resolution 0.05), recorded in millimeters and rounded to one decimal place. Each nisin producer was spotted in triplicate and overlaid with 3 separate cultures of each of the 18 *S. epidermidis* strains that is, biological repeats of all strains were tested. The results presented in Table 1 are the results obtained for one repeat but are representative of the results that were obtained for all three repeats.

### 4.3. Purification of Nisin A and The Derivatives M21V and M17Q

Nisin A, M17Q and M21V peptides were purified from overnight cultures of the relevant *L. lactis* strain as per the protocol set by Field et al. [42]. The strains were grown in 20 mL of GM17 broth, supplemented with chloramphenicol at 10 μg/mL, at 30 °C for 16 h and 1800 mL of sterile Tryptone Yeast broth was prepared to which 100 mL of a sterile 20% glucose solution (Sigma Aldrich Steinheim, Germany) and 100 mL of a sterile 20% β-glycerophosphate solution (Sigma Aldrich, Steinheim, Germany) were added to bring the final volume of broth to 2 L. 20 mL of GM17 overnight culture was used to seed the broth that is, a 1% inoculum. The strains were incubated at 30 °C for 18 h. After 18 h, the sample was then transferred to 500 mL centrifuge bottles and centrifuged at 7000× *g* for 15 min using a Sorvall RC 6+ model centrifuge. The supernatant and the cell pellet were separated, to extract nisin from both elements. The supernatant was filtered through a 30 cm long column, with an internal diameter of 2.5 cm, packed with 60 g of Amberlite XAD-16 beads (Sigma Aldrich, Steinheim, Germany) and prewashed with 1 L of sterile water. After passing the supernatant through the column followed by washing with 500 mL 30% ethanol, bound nisin was eluted using 400 mL 70% 2-propanol 0.1% trifluoroacetic acid. The bacterial cell pellet material was resuspended in 300 mL 70% 2-propanol 0.1% trifluoroacetic acid (TFA) and stirred at room temperature for 3 h. After the 3-h period, the cell pellet was centrifuged again at 7000× *g* for 15 min and the supernatant was retained. This supernatant was then combined with the eluted nisin from the column and placed into a rotary evaporator, (Buchi, Flawil, Switzerland). The 2-propanol was removed, concentrating the nisin in volume of approximately 300mL. The pH of the sample was adjusted to 4 and then applied to a 10 g (60 mL), SPE C-18 Bond Elute Column (Phenomenex, Cheshire, UK), pre-equilibrated with 60 mL methanol and 60 mL water and 120 mL of 30% ethanol was washed through the column prior to nisin being eluted into 60 mL of 70% 2-propanol 0.1% TFA. This 60 mL was separated into 15 mL samples, which were subjected to further rotary evaporation to reduce each fraction to a volume of 2 mL and 1.5 mL aliquots were run through a Phenomenex (Phenomenex, Cheshire, UK), C12 reverse phase (RP)-HPLC column (Jupiter 4u proteo 90 Å, 250 × 10.0 mm, 4 μm), equilibrated with 25% 2- propanol and 0.1% TFA. The nisin sample moved through the column at a flow rate of 1.2mL per minute. The antimicrobial fractions were pooled and then placed in the rotary evaporator to remove any remaining acetonitrile. The peptides were freeze dried, (using a FreezeZone 6 bench top freeze dryer) and stored at −20 °C.

### 4.4. Agar Well Diffusion Assays

10 mL of TSB was inoculated with a single *S. epidermidis* colony and cultured for 18 h at 37 °C. 20 mL volumes of molten TSA were inoculated with 200 μL of the overnight bacterial culture and poured into sterile Petri dishes (depth of agar was ~4 mm). A sterile glass pipette was used to create wells (diameter of 6 mm), in the agar. 30 μL of each purified peptide (Nisin A, M17Q and M21V, each at a concentration of 30 μM), was pipetted into individual wells. The plates were incubated upright for 18 h at 37 °C. Following the incubation period, any observed zones of inhibition were measured using calipers and recorded in millimeters. The Nisin A, M17Q and M21V peptides were tested against three biological repeats of the selected *S. epidermidis* strains.

### 4.5. Minimum Inhibitory Concentration (MIC) Assays

The MICs of peptides were determined as previously described [27]. MICs are broth-based assays that allow for clear confirmation of improved antimicrobial activity, as derivative peptides can appear artificially more effective than the wildtype due to better diffusion in agar based assays [24,25]. The assay was performed using flat bottom, non-tissue culture treated 96 well plates (Sarstedt, Nümbrecht, Germany). 200 μL volumes of PBS (Sigma Aldrich, Steinheim, Germany), containing 1% (wt/vol) Bovine Serum Albumin (Sigma Aldrich, Steinheim, Germany) was added to each well and incubated for 30 min at 37 °C. Wells were emptied then rinsed again with sterile PBS solution (Sigma Aldrich, Steinheim, Germany) and allowed dry. The wells were then filled with 100 μL of sterile TSB. The freeze-dried peptides were resuspended in TSB at a concentration of 30 μM and 100 μL of resuspended peptide was placed into the first well of the plate, before a twofold dilution was performed along the row of 12 wells. Strains *S. epidermidis* 28 and *S. epidermidis* 53, were grown overnight in 10mL of TSB. 400 μL of each overnight was sub-cultured in 10mL of fresh TSB and grown to an optical density of 0.5 at 600 nm (UV-1800 Shimadzu spectrophotometer). 20 μL of the culture was taken and inoculated into 980 μL of fresh TSB to obtain approximately 1 × 10^5^ cells per mL dilution was added into each well containing nisin peptides. The solution was pipetted up and down four times to ensure homogeneity. Once inoculated, the plates were incubated at 37 °C for 16 h. Following 16 h, the plates were visually inspected and the first clear well for each peptide was taken to be the MIC. The Nisin A, M17Q and M21V peptides were tested against the selected *S. epidermidis* strains in triplicate.

### 4.6. Kill Curve Assay

Ten mL of sterile TSB was inoculated with a single colony of the selected *S. epidermidis* strain. The broth was then incubated for 18 h at 37 °C. Ten μL of overnight culture was added to a sterile Eppendorf tubes containing 990 μL sterile TSB supplemented with either nisin A, M21V or M17Q peptide (final concentration of 3.75 μM (12.52 μg/mL)). Tubes were vortexed, (VELP Scientifica, Usmate Velate, Italy) and placed into a 37 °C incubator. After 1 h the Eppendorf tubes were removed from the incubator and 100 μL was taken and used to inoculate 900 μL of sterile ¼ strength Ringers.’ From this, a serial dilution was performed, vortexed and 100 μL of each dilution, (10^−1^ to 10^−5^), was plated onto TSA. The plates were incubated overnight at 37 °C after which they were counted and CFU/mL were calculated. The experiment was performed on three biological repeats of each strain (*S. epidermidis* 28 and *S. epidermidis* 53).

### 4.7. Biofilm Assays with Plastic Microtiter Plates

Biofilm formation on plastic surfaces was assessed as per the protocol established by Mathur et al. [28]. Ten mL of TSB supplemented with 1% glucose was inoculated with a single colony of the selected S. epidermidis strains and grown for 18 h at 37 °C. 200 μL of sterile TSB + 1% glucose was added to a single well to act as a sample blank and negative control. 3 different concentrations of nisin A, M21V or M17Q peptides were individually resuspended in sterile TSB + 1% glucose (the final volume of broth was 990 μL and the final concentrations of peptides were 1.875 μM (6.28 μg/mL), 3.75 μM (12.52 μg/mL) and 7.5 μM (25.04 μg/mL)). To each concentration of peptide, 10 μL of overnight *S. epidermidis* culture was added (to reach a final concentration of 4.23 × 10^7^ CFU/mL of *S. epidermidis* 28 and 3.11 × 10^7^ CFU/mL of *S. epidermidis* 53). Tubes were vortexed and 200 μL were transferred into individual wells of a 96 well plates (Sarstedt, Nümbrecht, Germany). The plates were incubated at 37 °C for 24 h. Each of the wells were washed gently with 200 μL of PBS three times (Sigma Aldrich, Steinheim, Germany). After washing, the cells were fixed to the plate by adding 200 μL of 2% sodium acetate (Sigma Aldrich, Steinheim, Germany). After removing the sodium acetate, 200 μL of 0.1% crystal violet (Prolab diagnostics, Bromborough, Birkenhead, UK), was added to each inoculated well and plates were left at room temperature for 10 min. The wells were rinsed with deionized water and allowed dry. When dry, the remaining crystal violet was resuspended using 200 μL of 95% ethanol (Sigma Aldrich, Steinheim, Germany). The absorbance reading of each well was taken using a calibrated Variskan Lux (Thermo Fischer, Darmstadt, Germany), A595nm. Nisin A, M17Q and M21V were tested against *S. epidermidis* 28 and 53 in triplicate, (i.e., three biological repeats).

### 4.8. Biofilm Assays on Stainless Steel

All square stainless steel coupons used in this test had an area 2 cm^2^. A modified version of the method employed by Minei et al. was used to analyze the formation of *S. epidermidis* biofilm on the stainless steel coupons [38]. Stainless steel coupons were sterilized by autoclaving at 121 °C, at 15 p.p.i for 15 min after which they were placed in flat bottomed 6 well plates (Costar, Analab Limited, Lisburn, Co. Antrim). Nisin A, M21V or M17Q peptides were individually resuspended in fresh, sterile TSB + 1% glucose in sterile Eppendorf tubes (the final volume of broth in each tube was 1.80 mL and the final concentration of each peptide was 3.75 μM (12.57 μg/mL). 20 μL of an overnight culture (grown in 10 mL of TSB supplemented with 1% glucose for 18 h at 37 °C) of the relevant *S. epidermidis* strain was added to each tube bringing the final volume up to 2 mL. Tubes were vortexed and the entire 2 mL of contents was aseptically added to a well containing a sterile steel coupon. A sterility control was created by incubating an autoclaved coupon in 2 mL uninoculated TSB + 1% glucose. The 6 well plates were incubated for 24 h at 37 °C. After incubation, each stainless steel coupon was carefully removed from the coupon using sterile forceps and dipped into a sterile container of autoclaved, distilled water to displace any planktonic cells. Then coupons were individually placed into sterile 50 mL falcon tubes with 5 mL of ¼ strength Ringers’ solution and vortexed for 30 s at 35 Hertz using tabletop VELP Scientifica vortex, to dislodge any biofilm into the surrounding solution. The plates were removed using sterile forceps and inspected. Any remaining biofilm was removed via scrubbing with a sterile cotton swab, which was rubbed vigorously along each side and surfaces of the coupon 20 times. The tip of the swab was cut and placed into the 10 mL solution of ¼ strength Ringers and biofilm prior to the solution being vortexed. One hundred μL of the solution was then taken and placed into 900 μL of sterile ¼ strength Ringers solution. A serial dilution was performed and each dilution (10^−1^ to 10^−6^), was plated onto TSA and grown overnight at 37 °C. Plates were counted and CFU/mL were calculated. Nisin A, M17Q and M21V were tested against *S. epidermidis* 28 and 53 in triplicate, using biological repeats of each strain.

### 4.9. Biofilm Assay on Various Catheter Materials

Three different catheter materials, (rubber, polyvinyl chloride and polyvinyl resin), were cut into pieces, (2 cm in length with diameters of 4 mm). The catheter pieces were soaked in a 2% glutaraldehyde solution for 30 min to sterilize them after which they were rinsed thoroughly in sterile water. Each piece was then placed in an individual, sterile, 2 mL microtube (Sarstedt, Nümbrecht, Germany). Nisin A, M21V or M17Q peptides were individually resuspended in 1.80 mL fresh, sterile TSB + 1% glucose (the final concentration of each peptide was 3.75 μM (12.52 μg/mL). The peptide supplemented broth was added to the tubes containing catheter pieces and tubes were incubated at 37 °C, shaking at 100 rpm (Titramax 100, Heidolph Instruments, Walpersdorfer, Schwabach, Germany), for 24 h. Following incubation, the catheter pieces were aseptically removed from the tubes in a biological safety cabinet and dipped slowly in a container of sterile distilled water to remove any planktonic cells. Once rinsed, the pieces were transferred to a new, sterile, 2 mL microtube containing 1 mL of sterile ¼ strength Ringers’ solution. Initially, samples were sonicated at 80% ultrasound power, using a desk top sonicating water bath at room temperature, (Transonic Digitals, Elma Ultrasonic, Singen, Germany) to detach the biofilm. The tubes were then vortexed at 40 hertz using a desk top VELP Scientifica desk top vortex, to loosen any remaining bound biofilm. When the biofilm was removed, 100 μL was taken from each tube, a serial dilution was performed and 100 μL of each dilution was plated onto fresh TSA plates. The plates were incubated for 18 h and CFU counts were carried out. This experiment was carried out in triplicate using three biological repeats of each *S. epidermidis* strain (*S. epidermidis* 28 and 53).

### 4.10. Testing the Efficacy of Nisin A and M17Q in a Simulated Wound Fluid

One hundred mL of a simulated wound fluid was prepared in accordance to the method used by Bradford et al. [44], and 3.3 g of bovine serum albumin, 0.58 g of sodium chloride and 0.5 g of calcium chloride (all purchased from Sigma Aldrich, Steinheim, Germany), were dissolved in 100 mL of distilled water. The fluid was filtered through a 0.45 μm filter, (Sarstedt, Nümbrecht, Germany). The simulated wound fluid was then aliquoted into 10 mL volumes and stored at 4 °C prior to use. Freeze dried nisin and nisin derivative peptides were resuspended in sterile TSB supplemented with 1% glucose at a concentration of 30 μM. 87.5 μL of reconstituted peptide and 655 μL of simulated wound fluid were placed into sterile 1.5 mL Eppendorf tubes and then inoculated with 7.5 μL of selected S. epidermidis overnight culture, (an initial inoculum of 3.8 × 10^7^ CFU/mL of *S. epidermidis* 28 and 4.6 × 10^6^ CFU/mL of *S. epidermidis* 53). This brought the final volume in each tube to 750 μL and the concentration of each nisin peptide was 3.75 μM (12.52 μg/mL). Tubes were vortexed and incubated for 1 h at 37 °C. One hundred μL was taken from each tube, a serial dilution was performed and 100 μL of each dilution was plated on fresh TSA plates. The plates were incubated for 18 h at 37 °C after which colonies were counted and CFU/mL calculated. Each *S. epidermidis* strains (28 and 53) was tested in triplicate that is, three biological repeats.

### 4.11. Reproducibility and Statistical Analyses

All experiments were performed in triplicate using three biological repeats. All CFU/mL data was converted to log_10_ prior to statistical analysis. All comparisons between data were based on the mean ± standard deviation. Statistical analysis was performed using MS Excel, version 16. In all cases, a *p* value < 0.05 was established as a threshold to consider a result as statistically significant. Asterisks indicating *, ** or *** designates statistically significant differences between samples (*p* < 0.05, *p* < 0.005 and *p* < 0.001, respectively).

## 5. Conclusions

In conclusion, in addition to providing further evidence that nisin A can inhibit *S. epidermidis* and impact biofilm formation, we report the identification of a novel bioengineered nisin A derivative M17Q with enhanced antimicrobial and anti-biofilm activity. In our opinion, these findings suggest that nisin and its bioengineered derivatives warrant further investigation as a potential alternative strategy for the control of *S. epidermidis*.

## Figures and Tables

**Table 1:** Comparison of the antimicrobial activity of wildtype nisin A (WT) producing *L. lactis* strain to that of 29 bioengineered nisin-derivative producing *L. lactis* strains through an agar based deferred antagonism assay. Each peptide producer was tested against 18 *S. epidermidis* strains in triplicate. The figures in the table are the results obtained for 1 repeat and are representative of the results obtained for all three biological repeats. Zone sizes are presented in millimeters. Values highlighted in bold indicate that the representative zone obtained from the bioengineered strain is larger than the zone obtained from the wildtype producer against that specific *S. epidermidis* strain.

*S. epidermidis* Strains																		
	**1**	**17**	**22**	**26**	**28**	**53**	**70**	**78**	**80**	**89**	**90**	**92**	**100**	**101**	**102**	**103**	**104**	**106**
**WT Nisin**	11	14.9	12.3	16.3	11.2	11	9.8	10.8	11.7	12	11	11	11	12.3	12.3	10.7	13.3	13.6
**Bioengineered** ***L. lactis* strains**																		
**M21V**	9.5	**15.9**	**13.7**	16	**12.7**	**12.6**	**13**	**12**	**13**	**13**	**12.7**	**12**	**13.1**	**13.7**	**13.3**	**13.6**	**14**	**14**
**M17Q**	**14.3**	**21**	**19.4**	**21**	**19.9**	**15**	**13.1**	**17.7**	**19.4**	**17.8**	**17**	**16.4**	**15.7**	**18.3**	**16.8**	**15**	**20.3**	**19.7**
**M21S**	**11.3**	**16.6**	**12.8**	**16.7**	**13.6**	**12.8**	**11**	**16.7**	**14.3**	**14.2**	**11.2**	11	**12.6**	**15.4**	**13**	**12.7**	**14.7**	**13.7**
**M21V-H31K**	9	**15.5**	**12.8**	15.7	**13**	**12.4**	9	**12**	**14**	11	**12**	**11**	11	12.1	**13.2**	**12.5**	13	12.7
**M21K**	10	13	9.8	15	**12**	10	**11.3**	**12**	10	**14.5**	**11.7**	9.7	**11.8**	**15**	12	**12.5**	**12.7**	12.8
**VGA**	**15.7**	**18.2**	**16.8**	**18**	**14.5**	**17**	**10.2**	**15.7**	**15.2**	**18**	**13.8**	**18**	**15.7**	**15**	**18**	**15**	**19.3**	**20.7**
**K12A**	10.7	13	**13.4**	15.9	**14**	**13.3**	**10.9**	10	**15**	**15.2**	**13.3**	**13**	**11.5**	**14**	**15**	**11.9**	**15.3**	**13.7**
**K12S**	10	**16**	12	**16.7**	**15**	**12**	8	**17**	**14**	**13.4**	**12.3**	**11.4**	**13**	**14.5**	**14.6**	**12.5**	**17.7**	**14.7**
**K12T**	10	**17.7**	10.5	15.1	10	10	8	10	9	10	**12**	9	11	10	**12.5**	**12.3**	**13.8**	11.3
**K22T**	10	**16.5**	11.2	**18.2**	**13.6**	**11.5**	**13.6**	**10.8**	**13.3**	**14.6**	**13.3**	10	**11.4**	**15**	**17**	**12.7**	**16**	**13.6**
**VGV**	10.1	14.9	**13.6**	**16.1**	**13.3**	**13.3**	**11**	9	**12.4**	8	**12**	**15.7**	11	**13.7**	11.2	**13.5**	**18.5**	**18**
**VGT**	**12.2**	**18**	**16.4**	**18**	**14.2**	**16**	**11**	**11**	**14**	**15.1**	**14.3**	**11.7**	**11.7**	**13.3**	**15.8**	**13**	**19**	**14.6**
**PIT**	**11.7**	**15.7**	11.3	**17**	**13**	**13.5**	**11**	**11**	10	**16.2**	**11.6**	10	**12.7**	11	**15.2**	**12.1**	**16.3**	**16.1**
**PGA**	**13**	**19.7**	**14.3**	**19.4**	**19.6**	**14**	**12.7**	**10.6**	**16.4**	9	**16.3**	**11.5**	**14.7**	**15**	**15.5**	**14.9**	**18**	**15.7**
**T2L**	**11**	10	9.5	12.2	10.5	10	8	**11.8**	10	12	**11.8**	**16**	**14.1**	10.7	**14.3**	**14**	**14.9**	**15.1**
**G18Dhb**	**12.2**	11	8	14.7	11	10.3	8	10	**16.3**	11.7	10	10	**12**	**13**	**12**	**12**	11.9	12.7
**SGK**	**13.4**	**17**	**13.7**	**18.8**	**15.3**	**13.7**	**10**	**11**	**13.7**	**13.7**	**15**	**13.4**	**14**	**13**	**13.7**	**13.7**	**16**	**16.5**
**PAQ**	10	12.5	11.7	**18**	**12**	**11.8**	**10**	**14**	9	**13**	**14**	**12**	**11**	**12.7**	**14.3**	**12.3**	**17**	**15.3**
**HTK**	**11.6**	**16.5**	10.4	**17**	**14**	10	**10**	**14.3**	**14.5**	**13.7**	**12**	**11.7**	**12.3**	**13**	**16**	**12.3**	**15.9**	**15.9**
**I4V**	0	14.6	11.7	**18**	**15.9**	**11.5**	9	**11**	10.7	**12.7**	**13**	**12**	**13**	11.7	**13.7**	**11**	**15**	**14.7**
**N2OP**	10	**15.5**	11.8	**18**	**14.5**	**11.3**	**10**	**12.3**	**16.9**	**14.7**	**16**	**13.6**	**11.5**	**15**	**13.7**	**13.5**	**16.9**	**16.4**
**S29R**	10	14	10.8	15.6	**12.6**	**11**	9	9	10.5	**14**	**12.7**	10	7	9	11.4	**11**	**13.4**	**14**
**S29E**	10	**15.4**	11.3	**16.7**	**11.7**	**11**	9	**11**	10	**13.6**	**11.7**	**11**	**11**	**12**	**17**	**16.9**	12	**14.2**
**S29A**	10	**16**	11.7	**17.3**	**14**	**11.4**	**10**	**11**	**11.6**	**14.7**	**12.7**	**12**	**11.9**	**13.6**	**14**	**13**	**15.3**	**14**
**S29D**	9	10.4	**14**	13.2	**11.9**	9.5	**11.5**	**11**	10.9	10	**11.3**	10.7	8	11	**14.3**	10	13	**12.2**
**M21A**	**12**	**17.4**	**18.3**	**19.3**	**14.4**	**14.7**	**11**	**11.5**	**17.3**	**15.5**	**14**	**16.7**	**15**	**15.5**	**15.2**	**13.2**	**16.7**	**16.7**
**H31K**	10.5	10.9	**14**	13	**14**	**11.3**	**10.1**	8.4	10	9.3	**11.9**	10.3	8	12	**12.5**	**10.4**	**12.7**	**14**
**AAK**	**12.5**	**16**	**13.2**	**15.7**	**11.3**	**11.4**	9	**12**	**12.5**	**15**	**13.3**	**13.8**	**12.3**	**13.7**	**16.3**	**12**	**13.7**	**15**
**AAA**	**15**	**19.9**	**21**	**21**	**20**	**14.6**	**15.9**	**17.5**	**16**	**19**	**15.8**	**17**	**16**	**19**	**19.3**	**14**	**20**	**19**

**Table 2:** Minimum inhibitory concentration (MIC) values for wild type peptide nisin A and its derivatives M21V and M17Q against *S. epidermidis*. All values in the Table are μM.

	Nisin A	M21V	M17Q
***S. epidermidis* 28**	3.75	1.875	1.875
***S. epidermidis* 53**	7.5	3.75	3.75

## Supplementary Materials

The following are available online at https://www.mdpi.com/2079-6382/9/6/305/s1. **Figure S1:** (**A**) Structure of the nisin peptide indicating the M17Q substitution (**B**) Amino acid sequence of nisin peptide and M17Q derivative peptide. **Figure S2:** Reversed–phase high performance liquid chromatography (RP-HPLC) profile for (A) derivative peptide M21V, (B) derivative peptide M17Q and (C) wildtype peptide nisin A. **Figure S3:** Chromatogram displaying MALDI-TOF Mass Spectrometry of lyophilized peptides, wildtype nisin A and derivative peptide M17Q.
