# Supplementary material for: Bioengineered Nisin Derivative M17Q Has Enhanced Activity against Staphylococcus epidermidis
Source: Antibiotics (Basel). 2020 Jun 6;9(6):305. doi: 10.3390/antibiotics9060305 (PMC7345907; doi:10.3390/antibiotics9060305)
Supplement: Supplementary file 1 [file antibiotics-09-00305-s001.pdf]

Supplementary Figures

A)

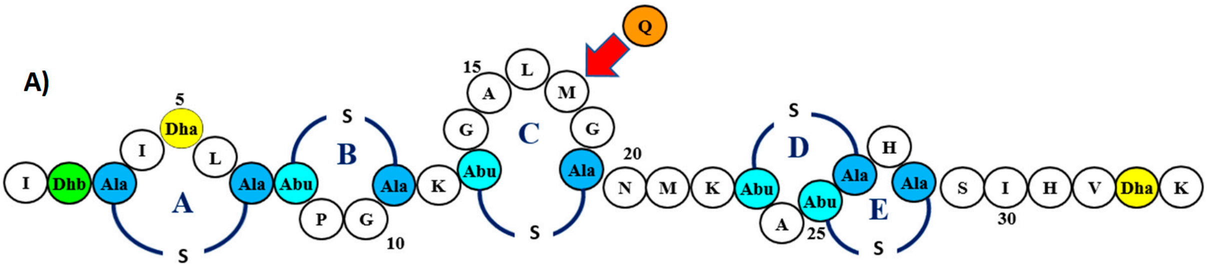

B)

Peptide Name	Amino Acid Sequence
**Nisin A (Wildtype peptide)**	ITSISLCTPGCKTGALMGCNMKTATCHCSIHVSK
**M17Q (Derivative peptide)**	ITSISLCTPGCKTGALQGCNMKTATCHCSIHVSK

Figure S1: A) The structure of the 34 amino acid nisin A peptide showing the location of the N-terminal domain, containing one lanthionine and two (β-methyl) lanthionine rings (A, B, and C) linked to the C-terminal intertwined rings (D and E) by a flexible hinge region. Standard residues are represented in the single letter code. The arrow indicates location of the methionine (M) to glutamine (G) substitution in the shortlisted nisin derivative peptide that is the focus of the current study i.e. the M that is at position 17 in the wildtype nisin peptide is replaced by a Q at position 17 in the derivative. The derivative is referred to as M17Q in the text.

B) The amino acid sequences of the wildtype peptide, nisin A and the bioengineered derivative peptide, M17Q, where ■ indicates the amino acid substitution at position 17. The enhanced derivative M17Q was identified from screening a bank of derivatives in which the codon for methionine at position 17 was randomized (i.e. M17X bank). Mass Spectrometry analysis of the enhanced nisin derivative producer revealed a mass of 3350.35 (see Figure S3). DNA sequencing of the nisin variant gene was also employed and revealed a codon change of ATG to CAG, confirming a methionine to glutamine (M17Q) substitution.

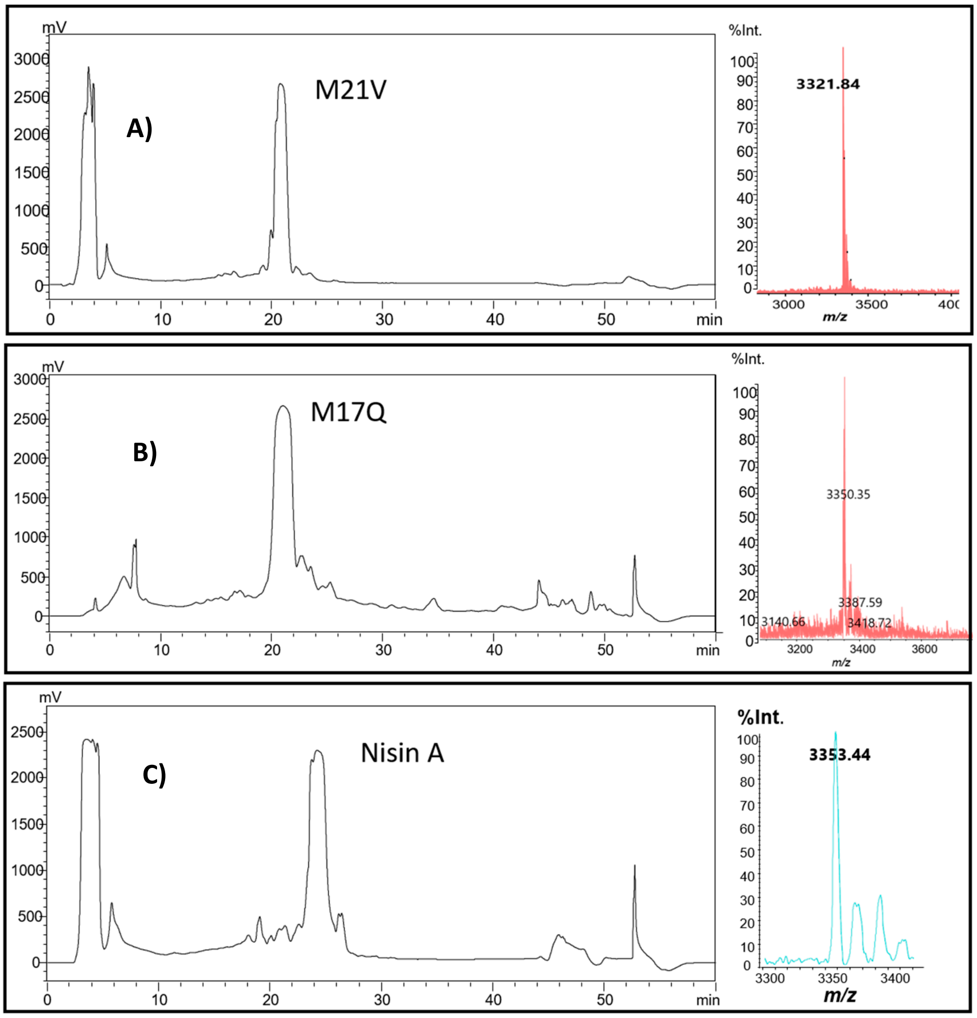

Fig. S2: Reversed–phase high performance liquid chromatography (RP-HPLC) profile for **A)** derivative peptide M21V, **B)** derivative peptide M17Q and **C)** wildtype peptide nisin A.

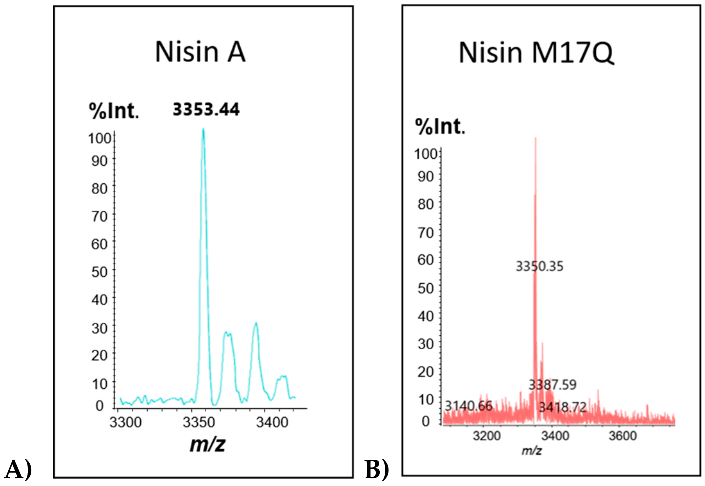

Fig. S3: Chromatogram displaying MALDI-TOF Mass Spectrometry of lyophilized peptides, wildtype nisin A and derivative peptide M17Q. The mass of **A)** the wildtype peptide was found to be 3353.44Da and **B)** the mass of the derivative peptide was found to be 3350.53Da.
